# Biliary reconstruction with or without an intraductal removable stent in liver transplantation: study protocol for a randomized controlled trial

**DOI:** 10.1186/s13063-015-1139-6

**Published:** 2015-12-30

**Authors:** Claire Goumard, Marine Cachanado, Astrid Herrero, Géraldine Rousseau, Federica Dondero, Philippe Compagnon, Emmanuel Boleslawski, Jean Yves Mabrut, Ephrem Salamé, Olivier Soubrane, Tabassome Simon, Olivier Scatton

**Affiliations:** Hepatobiliary surgery and liver transplantation, Assistance Publique-Hôpitaux de Paris (AP-HP), Hôpital Pitié-Salpétrière, UPMC-Paris 06, Paris, France; Department of Clinical Pharmacoloy, APHP, Hôpital St Antoine, Unité de Recherche Clinique de l’Est Parisien (URCEST), UPMC-Paris 06, Paris, France; Hepatobiliary surgery and liver transplantation, CHR Montpellier, Montpellier, France; Hepatobiliary surgery and liver transplantation, APHP, Hôpital Beaujon, Clichy, France; Hepatobiliary surgery and liver transplantation, APHP, Hôpital Henri Mondor, Creteil, France; Hepatobiliary surgery and liver transplantation, CHR Lille, Lille, France; Hepatobiliary surgery and liver transplantation, Hopital Edouard Herriot, Lyon, France; Hepatobiliary surgery and liver transplantation, CHR Tours, Tours, France

**Keywords:** Liver transplantation, Biliary complications, Biliary reconstruction, Intraductal stent

## Abstract

**Background:**

The incidence of biliary complications following liver transplantation (LT) remains high, ranging from 10 to 50 % of patients, especially when the diameter of the bile duct is smaller than 7 mm. Biliary reconstruction is most often performed by duct-to-duct anastomosis. In a preliminary study (n = 20), we previously reported a technique of biliary reconstruction using an intraductal stent tube followed by its endoscopic removal and showed both the feasibility and safety of this innovative procedure. The next step is to validate the potential benefit of this procedure in a randomized controlled trial.

**Design:**

This is a multicenter randomized controlled trial in France comparing the efficacy of biliary reconstruction with or without a removable intraductal stent on reducing biliary complications. Inclusion and randomization are performed during LT when a duct-to-duct biliary anastomosis smaller than 7 mm in diameter is envisioned. In the intraductal stent group, a custom-made segment of a T-tube is placed into the bile duct and removed endoscopically 4 to 6 months later. The surgical technique is described in a video during randomization and is available on the secure website used for inclusion and randomization. The primary endpoint is the occurrence of biliary complications, including biliary fistulae and strictures, during the 6 months of follow-up. Secondary evaluation criteria are the incidence of complications related to the stent placement and its extraction by endoscopy. The inclusion of 248 patients in total has been determined based on an expected incidence of biliary complications of 25 % in the non-IST group and a 60 % reduction of biliary complications (10 %) in the IST group.

**Discussion:**

Biliary complications following LT are significant causes of morbidity, retransplantation, and mortality. Although controversial, the use of a T-tube has been proven to be useless and even responsible for specific complications related to the external part of the tube in many studies, including several randomized trials. However, several studies have identified a small bile duct diameter as a risk factor for biliary stenosis. A threshold of 7 mm was found to be significantly associated with biliary stenosis. Our team published a preliminary study that included 20 patients using a new technique of intraductal stenting. Only four complications were reported in the overall study population, whereas no biliary complication occurred in the subgroup of patients who received a whole graft LT. Moreover, no technical failures and no procedure-related complications were noted before and during the drain removal. Although an intraductal stent tube in duct-to duct biliary anastomosis seems feasible and safe, a multicenter randomized controlled trial is needed to validate its benefit as a protective tool against the occurrence of biliary complications. One original aspect of this protocol is the video demonstration of the surgical procedure, which is available on the web to standardize and homogenize the technique. The surgical community may be inspired by this type of tool in the future to minimize technical bias related to technical issues.

**Trial registration:**

NCT02356939, date of registration 2 February 2015.

## Background

Biliary tract reconstruction during liver transplantation (LT) is the final technical step and the cornerstone of the procedure and is often performed by a duct-to-duct anastomosis. Rarely, recipient and donor biliary stump discrepancies or liver disease-related reasons could lead to a hepaticojejunostomy. The proper completion of this technical step is crucial for postoperative outcome [1, 2]. The incidence of biliary complications following liver transplantation (LT) remains high, ranging from 10 to 50 % of patients among clinical series despite an increasing experience worldwide [2–4]. These complications, mainly represented by biliary leaks and biliary strictures, are responsible for substantial post-transplantation morbidity and graft loss. Biliary leakage occurs mostly within 3 months postoperatively. This early complication reaches a 10 to 20 % incidence rate [3, 4]. Biliary strictures mainly occur later, within 5 to 8 months and up to 1 year in most. The reported incidence of biliary strictures still ranges from 5 to 30 % among large and recent clinical series [2, 4].

The use of an external T-tube to reduce biliary complications has been debated for decades [5–10]. The initial rationale was to facilitate biliary healing and maintain easy access to the biliary tract to perform a cholangiography until removal of the T-tube in consultation, in general within 6 weeks post-transplantation [11]. However, specific complications related to the cholangiography, such as cholangitis, and the development of endoscopic techniques, have reduced its legitimacy. Moreover, numerous published studies [5–8, 10], including three randomized studies [6–8], have shown not only an absence of difference of biliary complications with or without the T-tube use but also a specific morbidity related to the T-tube, such as cholangitis, and biliary leaks at the time of removal.

Those results have been criticized by two randomized studies [9, 12]. However, in one of these [9], the biliary strictures rate was similar between the T-tube and the non-T-tube group, and a higher general biliary complication rate (50 %) depended on an increased 14 % rate of pancreatitis. In the second study, which showed increased biliary strictures in the non-T-tube group in a monocentric design [12], the general biliary complications rate was similar, with a diagnosis of biliary stricture exclusively based on size discrepancy on imaging. The rate of Kehr-related complications was 23 % (13 % fistulae). The principal interest of this latter study was the significantly increased risk of biliary stricture in patients with bile duct size < 7 mm.

Based on these results, numerous teams do not use an external biliary drain in LT anymore. Moreover, the efficacy and safety of endoscopic biliary stenting has been highlighted in several studies in the treatment of biliary stenosis [13–17].

In this context, we developed a new technique of intraductal stent tube (IST) placement followed by its endoscopic removal in the 4 to 6 months postoperatively. The rationale was to prevent the biliary complications while avoiding the side effects related to an external T-tube use. We published a preliminary study conducted on 20 patients with a small graft bile duct (<5 mm) [18]. Biliary complications occurred in four patients, including one cholangitis, one hemobilia, one asymptomatic leakage, and one anastomotic stricture. No biliary complication occurred in the subgroup of patients who received a whole graft LT. No technical failure and no procedure-related complication were recorded during drain removal. Given the so–reported feasibility and safety of this innovative technique, we sought to evaluate its impact on biliary complications prevention.

The primary goal of this multicenter, randomized controlled study is to determine the incidence of biliary complications in the IST group, especially in case of small bile duct reconstruction, compared to the standard technique (duct-to-duct biliary reconstruction without IST) within the first 6 months post-liver transplantation. The second endpoint is to compare the incidence of complications related to the stent tube and its extraction by endoscopy.

## Methods

### Goals and outcomes

This multicenter randomized controlled study aims at comparing the efficacy of biliary intraductal stent tube placement in biliary end-to-end anastomosis to the standard technique (duct-to-duct biliary reconstruction without IST) on the incidence of biliary complications, that is, biliary strictures and biliary stenosis, within the first 6 months post-liver transplantation.

The primary outcome is the incidence of biliary strictures and biliary leakage within 6 months post-transplantation.

A biliary leakage was defined according to the ISGLS classification [19] as a bilirubin concentration in the drain fluid at least three times the serum bilirubin concentration on or after postoperative day 3 or as the need for radiologic or operative intervention resulting from biliary collections or bile peritonitis. A Grade A bile leakage caused no change in the patients' clinical management. A Grade B bile leakage required active therapeutic intervention but was manageable without relaparotomy, whereas in Grade C, bile leakage relaparotomy was required.

A biliary stenosis was defined by a size discrepancy between the two sides of the bile duct anastomosis on specific imaging (MR cholangiography or ERCP), associated with an upstream bile tract distention, with a clinical and biological cholestasis (gamma-glutamyl transferase and alkaline phosphatase three-fold elevation from baseline associated with an abnormal bilirubin serum level within 2 days), after excluding other cholestasis causes (rejection and viral reactivation).

Secondary endpoints are the incidence of specific complications related to the IST and its extraction by endoscopy: cholangitis, stent migration, extraction difficulties, acute pancreatitis, digestive perforation, and hemorrhage. The graft and patients’ survival at 6 months will also be analyzed as secondary endpoints.

### Patient selection

Patient inclusion is performed in consultation at the time of enlistment for LT, where they are informed and have to sign a written consent.

Inclusion criteria are as follows:

1. Adults older than 18 years-old.
2. Patients eligible for a liver transplantation.
3. Patients’ written informed consent signed.

Exclusion criteria include the following:

1. Biliary reconstruction requires a hepaticojejunostomy for anatomical/biliary disease reason.
2. Ineligibility for liver transplantation.

Definitive inclusion takes place in the operating room, during LT, and depends on the fulfillment of the following “definitive inclusion criteria”:

1. Duct-to-duct, end-to end biliary reconstruction.
2. Graft or recipient biliary duct diameter ≤ 7 mm.
3. Anatomically entire graft choledoque.
4. No graft from a circulatory arrested donor.

### Study course

The study is a multicenter, prospective, randomized controlled study. The follow-up is set at 6 months postoperatively to screen the majority of biliary complications. The inclusion period is set at 2 years, for a total study duration of 2.5 years.

The patient inclusion will be made in seven liver transplantation centers in France.

Definitive inclusion will take place in the operating room, during LT, and depends on fulfillment of the indicated “definitive inclusion criteria” (see above).

The randomization will be performed in the operating room by the investigator and coordinated by the Clinical Research Unit of the promoters’ center (Saint Antoine Hospital, Paris), with specific software accessible on the Internet.

**Fig. 1:**
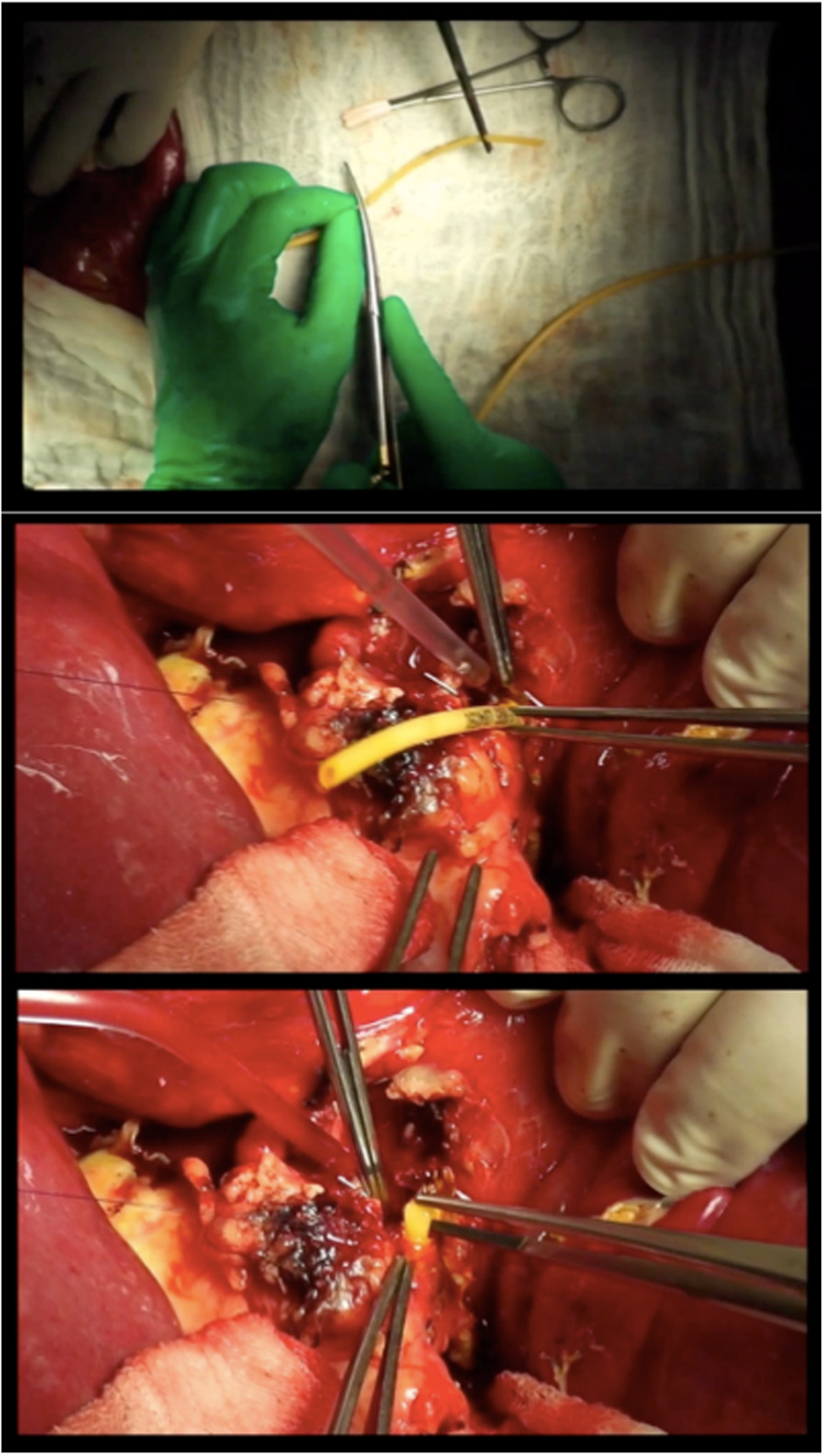
Intraductal stent tube insertion (short movie extracts)

In the IST group, the surgeon will place the IST in the bile duct, which is a custom-made segment (2 cm) of an 8 Charrière T-tube. The stent is inserted in the biliary duct without suture fixation (Fig. 1).

To minimize bias and homogenize the technique, a short technical explanatory movie was developed by the principal investigator’s team and provided on the internet. The technique of biliary reconstruction by either running suture or interrupted stiches is left to the surgeon preference because no impact has been demonstrated on the occurrence of biliary complications.

Each center performs its habitual postoperative follow-up. Clinical, biological, and radiological data are collected at Day 1, Day 7, Day 15, Month 1, Month 3, and Month 6. A Magnetic Resonance Cholangiography (MRC) is performed at 6 months post-LT, which is after the stent removal.

In the IST group, an endoscopic retrograde cholangio-pancreatography (ERCP) with sphincterotomy is planned between the 4^th^ and the 6^th^ month post-transplantation, requiring a short stay in the hospital, a general anesthesia, and clinical and biological tests including plasmatic lipase dosage at Day 1.

Every undesirable event will be immediately reported to the sponsor for further investigation of its severity. Severe undesirable events are previously defined.

### Statistical analysis

The inclusion of 248 patients in total has been estimated based on an expected incidence of biliary complications of 25 % in the non-IST group, and a 60 % reduction of biliary complications (10 %) in the IST group with an alpha risk of 5 % and a beta risk of 20 %.

The statistical analysis will be in an intention-to-treat fashion. The frequency of occurrence of a biliary complication will be compared between groups with a χ^2^ test or a Fisher test as appropriate.

Adjusting factors such as arterial thrombosis or surgical technique will be managed with logistic regression models as appropriate.

### Ethical approval

This trial has been approved by the *Comité de Protection des Personnes (CPP)* Ile de France III - 3170 (File ref: 2014-A00866-41).

## Discussion

Despite a global improvement in overall survival rates in LT in the last decades, decreasing the incidence and severity of biliary complications remains a major goal for transplant surgeons.

External drainage using T-tube has not proven its efficacy in a consensual way. Still debated, T-tube placement is not used anymore in numerous teams because of the nonproven preventative effect on biliary complications and because of the specific complications related to its use and removal, such as bile leaks and cholangitis [5–8, 10]. Moreover, such a hypothetic prevention of biliary complication is not supported by the fact that T-tubes are removed early, within the initial 2 months postoperatively. This delay is probably too short to prevent the development of a biliary stricture. This lack of long-term effect may be advantageously cleared by the IST technique. This innovative, easy to reproduce technique allows a longer stenting effect on biliary reconstruction and avoids specific morbidity related to the external biliary drainage [18].

Specific complications related to endoscopic extraction of the stent and ERCP with sphincterotomy, in general, may be expected in this study. However, large clinical studies report very low complication rates; in the study of Cotton et al. (2009), in an analysis of more than 11,000 procedures, the overall complication rate was about 4 %, with very low incidence of hemorrhage (0.3 %) or acute pancreatitis (2 to 3 %) [20]. In our preliminary study carried out on 20 patients, no technical failures and no procedure-related complications were recorded during drain removal. A limited sphincterotomy was performed in 17 patients of 20 without any complication [18]. The small number of patients and the single-center referring to an experienced endoscopist may also explain these results.

Some complications specific to the stent may also be expected, such as stent migration in the duodenum, infectious cholangitis, or extraction difficulties due to stent incrustation. In our experience, one cholangitis occurred and was successfully treated by antibiotics and drain removal. This latter patient had a transpapillary drain that may explain the occurrence of a backflow into the biliary tract [18]. Consequently, we decided to systematically place the tube above the ampulla of Vater. One patient experienced a clinical asymptomatic spontaneous migration of the stent in the duodenum, without any consequences. No difficulties were recorded during IST ablation because of stent incrustation, event after more than a 6-month postoperative delay.

We previously performed this technique in patients with a graft bile duct diameter less than 5 mm. This option was based on several studies [12, 21–23] that demonstrated a significant association between the risk of biliary stenosis and the small diameter of the biliary duct. Asian studies [21–23] found a significantly increased risk for less than 5 mm bile duct diameter, but these studies are conducted on reduced liver grafts obtained from living donors. A recent trial [12] found a significantly increased risk for bile ducts less than 7 mm in diameter in 197 transplanted patients from whole cadaveric liver grafts. Thus, a cut-off value of 7 mm for graft bile duct diameter was chosen for this trial.

In the design of this trial, we assumed an expected biliary complication rate in the non-IST group of 25 %. We considered that these patients with a small bile duct diameter (<7 mm) presented a higher risk of biliary strictures, according to the literature. A 60 % reduction in biliary complications, representing a 10 % biliary complication rate in the IST group, seemed to be a reasonable goal.

To avoid any bias related to technical issues and given the multicenter design of the trial, we decided to produce a movie that clearly explains the procedure step-by-step. To our knowledge, this is the first time that such a technical standardization has been used in the surgical research field, especially its accessibility on the Internet, which means it always available during the randomization in the operating theatre. This technique will be available on a secure website that is already being used for inclusion and randomization, and later will be attached to the publication of the results. This innovative form of surgical diffusion will be useful to avoid technique bias and to standardize the technique worldwide when published.

In conclusion, this randomized, multicenter controlled trial aims at validating this technique as a safe, innovative way to prevent biliary complications in high-risk patients following liver transplantation.

## Trial status

The proposed trial has been validated and accepted for financial support by the French National Health Ministry (Programme Hospitalier de Recherche Clinique (PHRC AOR13028 – P130919)). Patient inclusions have been ongoing since 30 May 2015.

## Abbreviations

ERC: endoscopic retrograde cholangiography
IST: intraductal stent tube
LT: liver transplantation
MRC: magnetic resonance cholangio-pancreatography
